# Public Health benefits by implementing digital symptom diaries for COVID patients from Cologne

**DOI:** 10.1093/eurpub/ckac131.049

**Published:** 2022-10-25

**Authors:** B Gruene, S Kugler, A Kuefer-Weiß, A Wolff, A Kossow, J Nießen, F Neuhann, S Ginzel, M Buess

**Affiliations:** 1 Health Department Cologne, Cologne, Germany; 2 Fraunhofer Institute for Intelligent Analysis, Sankt Augustin, Germany; 3 Institut for Global Health, University of Heidelberg, Heidelberg, Germany; 4 Institut for Hygiene, University of Muenster, Muenster, Germany

## Abstract

**Background:**

High rate of people infected with SARS-CoV-2 and their contacts in Cologne, Germany required innovative tools for notification, monitoring and reporting. The digital tool for COVID19 (DiKoMa) provides self-service symptom diaries allowing (a) the stratification for prioritized telephone contact by the health authority and (b) training a machine learning (ML) model that predicts infections with prevailing dominant variant (PDV) from early symptom profiles (SP).

**Methods:**

Pseudononymized SP covering the first week of diary recordings were included for training (16646 index, 11582 contacts). A balanced random forest (BRF) model was trained to differentiate early predictive symptom patterns of different PDV and contact persons. Model evaluation was performed using sex and age stratified cross validation (CV), the model was validated on SP recorded from days 1 and 6.

**Results:**

From 03/20 to 02/22, 90478 indeces and 75444 contact persons reported symptoms and health status, covering 46% and 42% of all reported cases, respectively. Diaries contained between 1-52 entries (566791, median 2). Daily analysis of entries, prioritized according to age, prevalent co-morbidities and detoriation of symptoms allowed risk adjusted follow up even during phases with high case notification rates. The top 5 predictive factors of the BRF were immunization, cough, dysgeusia and dysnosmia, fatigue, and sniffles to differentiate infection between wildtype, three PDV and contact persons (CV AUC 80.6%, Validation AUC 77.1%).

**Conclusions:**

The use of digital symptom diary surveillance helps to provide appropriate medical support for patients on a large scale. Machine learning shows potential for symptom based risk assessment to differentiate PDV for future outbreaks and can thus become a valuable tool alongside specific laboratory diagnostics.

**Key messages:**

• Digital symptom diaries are a powerful and widely accepted tool to attend COVID19 patients in isolation. They allow risk stratification for follow up and are a low-threshold service.

• Machine learning supports index case identification by symptom analysis and can thus become a valuable tool alongside specific laboratory diagnostics.

